# Effects of processing on carbendazim residue in *Pleurotus ostreatus*

**DOI:** 10.1002/fsn3.328

**Published:** 2016-02-24

**Authors:** Erdong Xia, Wuqun Tao, Xi Yao, Jin Wang, Feng Tang

**Affiliations:** ^1^ International Center for Bamboo and RattanBeijing100102China

**Keywords:** Carbendazim, dissipation, pesticide residue, *Pleurotus ostreatus*, processing

## Abstract

Samples of *Pleurotus ostreatus* were exposed to fungicide carbendazim to study the effect of processing on the residues. In most cases, processing operations led to a significant decrease in residue levels in the finished products, particularly through washing, drying, and cooking processes. The results indicated that rinsing under running tap water led to more than 70.30% loss in carbendazim residues. When dried under sunlight could remove more than 70.30% residues. There was a 63.90–97.14% reduction after steaming, with processing time extending, the removal rates increased especially for lower initial residue level samples. The residue was almost completely removed by frying combined with microwave heating. Furthermore, boiling the mushrooms reduced the residue in the mushroom and no carbendazim residues were determined in the broth.

## Introduction

Mushrooms have been eaten and appreciated for their flavor, economical and ecological values, and medicinal properties for many years. The production of mushrooms is regarded as the second most important commercial microbial technology next to yeast (Pathak et al. 2009). Some mushrooms can be cultivated easily and have significant worldwide markets. The production of mushrooms in 2011 was 25.71 million tons, including 5.63 million tons of *Pleurotus ostreatus*, in China (Wu et al. 2013). *Pleurotus ostreatus* is the second most cultivated edible mushroom worldwide after *Agaricus bisporus*. It has economic and ecological values and medicinal properties (Carmen 2010). However, at cultivation stage, preventive treatments with fungicides and insecticides are usually carried out to prevent fungal and insect proliferation that may contaminate the mushroom. Furthermore, the inadequate quality of cultivated substrate, such as grain or grain mixed with grain straw, and irrigation water can also represent a hazard for the cultivation (José et al. 2013).

Carbendazim, a broad spectrum fungicide, is widely used in the production of edible mushroom to prevent fungal proliferation and was detected in the mushrooms occasionally, which involve possible health risk and environmental pollution (Dong et al. 2010).

Concerning human health, scientists and food processors have long been interested in the effect of processing on pesticide residues in food. Food processing treatments such as washing, peeling, canning, or cooking lead to a significant reduction in pesticide residues. Kaushik et al. (2009) reviewed the common food processing operations along with the degree of residue removal in each process, which include baking; bread making; dairy product manufacture; drying; thermal processing; fermentation; freezing; infusion; juicing; malting; milling; parboiling; peeling; peeling and cooking; storage; storage and milling; washing; washing and cooking; washing and drying; washing and peeling; washing, peeling, and juicing; and wine making (Kaushik et al. 2009). Extensive literature demonstrates that in most cases processing leads to large reductions in residue levels in the prepared food, particularly through washing, peeling, and cooking operations (Elkins 1989; Chin 1991; Farris et al. 1992; Holland et al. 1994; Cabras and Angioni 2000; Ma et al. 2005; Hassanzadeh et al. 2010). A study on the degradation of Azinphos‐methyl in fruits showed that residues were detected in apple juice but not in lemon juice (Athanasopoulos and Pappas 2000). However, the effect of processing on some economically important edible mushrooms is less known.

The main objective of this work is to investigate the effect of processing on residues in *P. ostreatus* after exposing of carbendazim. This will offer a suitable means to tackle the current scenario of unsafe food at domestic and industrial level, especially in developing countries. In addition, it will guide the producer to application of authorized phytosanitary treatments to ensure the quality of their productions in the cultivation line of *P. ostreatus* and other cultivated edible fungi.

## Materials and Methods

### Materials

Carbendazim standard (99.7% purity) was obtained from Sigma‐Aldrich (St. Louis, MO, US). The liquid detergent for fruits and vegetables was from Beijing Goldfish Technology Co., Ltd. (Beijing, China) and carbendazim 50% wettable powder from Jiangyin Fuda Agrochemicals Co., Ltd (Jiangyin, Wuxi, Jiangsu Province, China).

All reagents, including ethyl acetate, sodium chloride, and sodium sulfate anhydrous, were of analytical grade. Methanol and acetonitrile were high‐performance liquid chromatography grade.

### Sample collection

*Pleurotus ostreatus* mushroom sample was purchased from the supermarket. The mushroom was randomly divided into two subgroups such that one subgroup was dispatched for analysis to certify as pesticide‐free, while the other set of samples was exposed to commercial carbendazim formulations and subsequently processed. The samples were maintained at 4°C and analyzed within 24–48 h of purchase; otherwise, they were placed unchopped in polyethylene bags, labeled, and kept at −18°C.

### Exposure to carbendazim in the laboratory

*Pleurotus ostreatus* mushroom samples were dipped in the laboratory with aqueous mixtures of the commercial formulations of 50% carbendazim wettable powder (WP). The aqueous mixtures of the fungicide were prepared in distilled water according to the pre‐experimental conditions, to make the residues in mushroom around at 10 mg kg^−1^, 1 mg kg^−1^, and 0.1 mg kg^−1^, respectively. The exposure to carbendazim was achieved by dipping the residue‐free mushroom samples in the aqueous mixtures for 2 min. The samples were then kept at room temperature in a fume cupboard for 4 h allowing for attachment and penetration of the fungicide into the mushroom.

### Processing of *P. ostreatus* mushroom

All mushroom samples were processed in the laboratory. Samples taken at each of the processing steps consisted of five replicates of 100 g each.

#### Drying

Two methods were used for drying. A portion of *P. ostreatus* samples were placed in clean plates and dried under sunlight, while the other portion were placed in the freeze drying flask and dried under −86°C with a high vacuum of 0.06 mbar.

#### Washing

Three washing modes were used. One portion of mushroom was washed by placing it in a plastic colander and rinsed under running tap water for 5 min, with gentle rotation by hand. Another portion was washed with detergent aid and done in accordance to its instructions, and the last part was soaked with tap water for 10 min. After washing, the surface water of samples was blotted up with the filter paper.

#### Cooking

The samples were sliced into small pieces about 5 cm × 1 cm in length and width, and then cooked as following:

##### Frying

A portion of 100 g sliced samples were put into the oil pan with 300 mL soybean oil at about 220°C to fry for 5 min or 7 min, respectively. Then, the samples were taken out, cooled, and measured.

##### Boiling

The samples were put into the pot with 2 L boiling water, and cooked for 10 min covered with or without lid, respectively. Then, the samples were taken out, cooled, and measured. The residues of the mushroom and the broth were determined, respectively.

##### Steaming

The sliced mushroom was steamed for 10 min and 15 min, respectively. Then, the samples were taken out, cooled, and measured.

##### Microwave heating

A portion of fried mushroom was cooled to room temperature, and then kept in a microwave oven to heat for 2 min.

### Determination of carbendazim residue

The fungicide residue was analyzed by the ethyl acetate solvent extraction followed by determination with high‐performance liquid chromatography coupled with an ultraviolet detector (HPLC‐UVD). The samples were chopped and homogenized in a food chopper. About 10 g portions were weighed and blended with 80 mL ethyl acetate and 5 g sodium chloride. The extract was filtered through filter paper with a layer of 20 g anhydrous sodium sulfate on top and rinsed twice with 20 mL ethyl acetate. Extraction was repeated with another 60 mL ethyl acetate. The combined extracts were concentrated just to dryness using a rotary vacuum evaporator at 40°C, then redissolved in 10 mL methanol, and filtered through a 0.45 *μ*m filter membrane before HPLC determination. The residue was analyzed by HPLC with variable wavelength UV detector. An Agilent 4.6 mm × 250 mm Tc‐C18 column was used. The mobile phase consisted of acetonitrile (A) and water (B). Elution was performed in the gradient mode; the initial ratio was 20% A to 80% B, 5 min later it changed to 50% A and 50% B and last for 10 min. The flow rate was 1 mL min^−1^ and injection volume was 20 *μ*L. The temperature of column was 30°C.

## Results and Discussion

### Recovery and LOD of carbendazim in the mushroom

Recovery experiments were performed to validate the determination method. Blank samples were fortified with the fungicide at three fortification levels. For raw mushroom, the mean recoveries ranged from 87.60% to 106.82% with relative standard deviation (RSD) 0.96–2.63%. The recoveries of fried mushroom were 75.0–89.96%, and the RSD were between 4.74% and 10.76% (Table 1). The limit of detection (LOD) was 0.03 mg kg^−1^. In conclusion, the methods provide a determination of carbendazim residues in raw and cooked *P. ostreatus* samples, with acceptable recovery and repeatability, and sufficient limit of determination.

**Table 1:** Recoveries of carbendazim in *Pleurotus ostreatus* and cooked mushroom (*N* = 5)

Sample	Fortified level (mg kg^−1^)	Found level (mg kg^−1^)					Recovery (%)	RSD (%)
Raw mushroom	0.1	0.086	0.086	0.089	0.091	0.086	87.60	2.63
	1.0	1.04	1.06	1.06	1.08	1.10	106.82	2.43
	10.0	9.66	9.54	9.54	9.72	9.51	95.96	0.96
Cooked mushroom	0.1	0.065	0.070	0.078	0.079	0.083	75.00	9.75
	1.0	0.738	0.810	0.847	0.973	0.782	83.00	10.76
	10.0	8.40	9.30	9.50	8.84	8.93	89.96	4.74

RSD, relative standard deviation

### Effects of washing on carbendazim residues in *P. ostreatus*

The significant effect of washing on carbendazim residues is shown in Table 2. The greatest impact in the carbendazim residues was when rinsed under running tap water. The dissipation rate is more than 70.30%. When the mushroom samples were soaked in tap water, 35.64–93.61% of residues were removed. However, washing with detergent did not showed aid to remove the residue, and the fortified levels in the starting material were dropped by 62.38–87.73%. The claim that “the detergent removes 98% more pesticide residue than water alone” is not supported by the results. The effectiveness of a water rinse to remove trace surface residues invalidates the detergent claims for produce rinses.

**Table 2:** Effects of washing on carbendazim residues in *Pleurotus ostreatus*

Washing methods	Before washing (mg kg^−1^)	After washing (mg kg^−1^)	Removal rate (%)
Soaked in tap water	0.101	0.065 ± 0.0079	35.64 ± 7.82
	1.05	0.148 ± 0.0110	85.90 ± 1.05
	7.51	0.480 ± 0.0598	93.61 ± 0.80
Rinsed under running tap water	0.101	<LOD	>70.30
	1.05	0.153 ± 0.0069	85.43 ± 0.66
	7.51	0.523 ± 0.0426	93.04 ± 0.57
Tap water with detergent aid	0.101	0.038 ± 0.0038	62.38 ± 3.76
	1.05	0.221 ± 0.0303	78.95 ± 2.89
	7.51	0.922 ± 0.0179	87.73 ± 0.24

### Effects of drying on carbendazim residues in *P*. ostreatus

Drying is the oldest method of preserving food. As compared with other methods, drying is quite simple. Food can be dried in several ways, for example, under the sun or in an oven or a food dryer can also be used.

Table 3 shows that the amount of carbendazim in the mushroom showed an apparent decrease after drying. Carbendazim residues of freeze‐dried mushroom were higher than that dried under sunlight. No residues were detected in finished dried products at initial levels of 0.101 mg kg^−1^ and 1.050 mg kg^−1^ after dried under sunlight. That inferred high temperature and ultraviolet may accelerate the degradation and/or volatile nature of carbendazim residues in mushroom.

**Table 3:** Effects of drying on carbendazim residues in *Pleurotus ostreatus* (*N* = 5)

Drying methods	Before drying (mg kg^−1^)	After drying (mg kg^−1^)	Removal rate (%)
Dried under sunlight	0.101	<LOD	>70.30
	1.05	<LOD	>97.14
	7.51	0.424 ± 0.0311	94.36 ± 0.41
Freeze dried	0.101	<LOD	>70.30
	1.05	0.062 ± 0.0057	94.10 ± 0.54
	7.51	1.789 ± 0.2180	76.19 ± 2.90

LOD, limit of detection.

Drying has been found to reduce the pesticide residues considerably. For example, drying of grapes lead to 64.2–71.9% losses of methamidophos possibly due to evaporation of the pesticide during the process (Athanasopoulos et al. 2005).

### Effects of cooking on carbendazim residues in *P. ostreatus*

Generally mushrooms are consumed after cooking in Chinese cuisine. The effect of cooking on carbendazim residues in mushroom is shown in Tables 4 and 5. The reduction of carbendazim in *P. ostreatus* is obvious by various processing methods. There was a 24.75–98.26% reduction after frying for 5 min. With processing time extending, the removal rates increased especially for lower initial residue level sample. This accumulated to about 100.00% reduction after microwave heating. Similar results were found in the steamed mushroom samples. For lower residue level sample, steamed for 10 min, the remaining residue was below LOD (0.03 mg kg^−1^). The carbendazim residues in boiled mushroom and its broth were also detected to study the dissipation and transferring from mushroom to the broth. Results showed that removal rates of carbendazim boiled with covered lid were lower than that without lid. This may because it is easier for carbendazim volatile without covered lid. The residues detected in the broth were below LOD (Table 5).

**Table 4:** Effects of cooking on carbendazim residues in *Pleurotus ostreatus* (*N* = 5)

Cooking methods	Before cooking (mg kg^−1^)	After cooking (mg kg^−1^)	Removal rate (%)
Steamed for 10 min	0.101	<LOD	>70.30
	1.05	0.379 ± 0.0501	63.90 ± 4.77
	7.51	0.725 ± 0.1491	90.38 ± 1.98
Steamed for 15 min	0.101	<LOD	>70.30
	1.05	<LOD	>97.14
	7.51	0.487 ± 0.0232	93.5 ± 0.31
Fried for 5 min	0.101	0.076 ± 0.0030	24.75 ± 2.97
	1.05	0.098 ± 0.0036	90.67 ± 0.34
	7.51	0.131 ± 0.0072	98.26 ± 0.10
Fried for 7 min	0.101	0.057 ± 0.0069	43.56 ± 6.83
	1.05	0.072 ± 0.0014	93.14 ± 0.13
	7.51	0.080 ± 0.0083	98.94 ± 0.11
Microwave heating after fried	0.101	<LOD	>70.30
	1.05	<LOD	>97.14
	7.51	0.049 ± 0.0048	99.35 ± 0.06

LOD, limit of detection.

**Table 5:** Effects of boiling on carbendazim residues in the mushroom and the soup (*N* = 5)

Boiling methods	Before boiling (mg kg^−1^)	After boiling (mg kg^−1^)	Removal rate (%)
Boiled with lid covered	0.101	0.075 ± 0.0232	25.74 ± 2.28
	1.05	0.462 ± 0.0061	56.00 ± 0.58
	7.51	0.309 ± 0.044	95.89 ± 0.59
Boiled without lid	0.101	<LOD	>70.30
	1.05	0.098 ± 0.0020	90.67 ± 0.19
	7.51	0.605 ± 0.0531	91.95 ± 0.71
Boiling of the soup with lid covered	0.101^a^	<LOD	–
	1.05^a^	<LOD	–
	7.51^a^	<LOD	–
Boiling of the soup without lid	0.101^a^	<LOD	–
	1.05^a^	<LOD	–
	7.5^a^	<LOD	–

a Residue level of mushroom before boiling.

LOD, limit of detection

Cooking operation reduced the pesticide residue was reported by other scientists. Frying affected organophosphorus residues more than organochlorine as the percent reduction of organophosphorus ranged between 49% and 53%. The level of reduction ranged between 30.1% and 35.3% for the organochlorines (Soliman 2001).

## Conclusions

The experiments on the effect of food preparative operations on carbendazim residues have demonstrated that processing remove large amounts of the carbendazim residues present on the mushroom. The washing, drying, and cooking of mushrooms lowered the carbendazim levels considerably, from 60% to 99.35% in most cases. No carbendazim residues were determined in the broth after they were boiled. The overall results can reassure the consumer that cooked mushrooms are wholesome and safe.

## Conflict of Interest

None declared.
